# The 2021 AHA/ACC/SCAI Coronary Artery Revascularization Recommendations: Need for Emphasis on Prevention and Future Considerations

**DOI:** 10.1016/j.jacadv.2022.100006

**Published:** 2022-03-16

**Authors:** Rhanderson Cardoso, Arielle Abovich, William E. Boden, Armin Arbab-Zadeh, Ron Blankstein, Roger S. Blumenthal

**Affiliations:** a Cardiovascular Division, Brigham and Women’s Hospital and Harvard Medical School, Boston, Massachusetts, USA; b Johns Hopkins Ciccarone Center for the Prevention of Cardiovascular Disease, Johns Hopkins University School of Medicine, Baltimore, Maryland, USA; c VA New England Health Care System, Massachusetts Veterans Epidemiology, Research, and Informatics Center, Boston University School of Medicine, Boston, Massachusetts, USA

**Keywords:** coronary artery disease, guideline-directed medical therapy, revascularization

For nearly 4 decades, controversy persists about the management of patients with coronary artery disease (CAD) among cardiologists and cardiac surgeons regarding the optimal approach to myocardial revascularization, as both catheter-based and surgical techniques have continued to evolve. Likewise, in recent years, there also have been profound developments of increasingly more effective secondary prevention strategies directed at multiple therapeutic targets of residual cardiovascular risk including serum lipids, blood pressure, platelet function, and vascular inflammation which, in the aggregate, provide a robust medical therapy platform for enhancing improved outcomes.

Against this backdrop, the recent publication of the 2021 American Heart Association (AHA)/American College of Cardiology (ACC)/Society for Cardiovascular Angiography and Interventions (SCAI) Guideline for Coronary Artery Revascularization^1^ affords us important new therapeutic insights and treatment recommendations. We commend the guideline committee for their formidable accomplishment that provides clinicians with increased clarity and guidance for when, and in whom, surgical or percutaneous revascularization should be strongly considered (Class I and IIa) or withheld (Class III) or when these procedures are of uncertain benefit (Class IIb). However, we believe it is time for the academic and practicing cardiology community to place a greater focus on how multifaceted, preventive pharmacotherapy has become an indispensable component of modern-day care for patients with established CAD to improve clinical outcomes.

## Medical therapy reduces events for most patients with CAD

The results from the ISCHEMIA (the International Study of Comparative Health Effectiveness With Medical and Invasive Approaches) and COURAGE (Clinical Outcomes Utilizing Revascularization and Aggressive Drug Evaluation) trials clearly demonstrated that a strategy of routine revascularization in patients with stable CAD provided no incremental benefit in reducing cardiovascular death or myocardial infarction when added to guideline-directed medical therapy (GDMT), which included robust pharmacologic secondary prevention combined with intensive lifestyle intervention during long-term follow-up. This was true even in the presence of substantial baseline ischemia, with the notable exception of patients with impaired systolic function or left main coronary artery disease who were not included in the trials^2^, ^3^, ^4^ (see Table 1).

**Table 1:** Optimal Preventive Pharmacotherapy for Patients With Established CAD Being Considered for Coronary Revascularization

Medical Therapy	Randomized Controlled Trials	ACC/AHA Recommendations^14^, ^15^, ^16^	ESC Recommendations^17^
Hypertension	∼25% relative risk reduction with intensive BP treatment (goal <120 mmHg) relative to a standard treatment with goal <140 mmHg in patients with increased CV risk.^18^	Use BP-lowering medication for secondary prevention of CVD in patients with an average SBP ≥130 mmHg (Class I).	In patients with CAD, the recommended office systolic blood pressure target range is 120 to 130 mmHg (Class I).
Statin therapy	High-intensity statins ↓LDL-C ≥50%; 1 mmol/L (38 mg/dL) ↓ LDL-C ↓MACE ∼20%-25%	Goal LDL-C < 70 mg/dL and ≥50% reduction in baseline LDL-C (Class I).	LDL-C goal <55 mg/dL and ≥50% reduction from baseline (Class I).
Nonstatin therapy	1. Ezetimibe: ↓LDL-C ∼25%; 2. PCSK9 inhibitors: ↓LDL-C ∼60%; 3. Bempedoic acid: ↓LDL-C 15%-20%; pending outcome trials	In very-high-risk patients with LDL-C ≥70 mg/dL despite maximally tolerated statin, add ezetimibe (class IIa); add PCSK9 inhibitor after ezetimibe if LDL-C remains ≥70 mg/dL (Class IIa)	If LDL-C goals are not achieved with a statin, add ezetimibe (class I); add PCSK9 inhibitor if still not at goal despite statin and ezetimibe (Class I).
Icosapent ethyl	Icosapent ethyl reduced cardiovascular death, MI, stroke, coronary revascularization, or unstable angina by 25% among patients with established CVD and a fasting triglyceride level of 135 to 499 mg/dL.^19^	May consider icosapent ethyl in patients with clinical ASCVD or diabetes and fasting triglycerides between 150 and 499 mg/dL, if LDL-C is at goal.^a^	In patients with established ASCVD with triglycerides >135 mg/dL despite statin treatment and lifestyle measures, icosapent ethyl may be considered in combination with a statin (Class IIb).
ACE inhibitors or ARB	Reduce the risk of overall and cardiovascular death, MI, and stroke in patients with CAD.^20^	Recommended if a patient has heart failure, hypertension, or diabetes (Class I).	Recommended if a patient has heart failure, hypertension, or diabetes (Class I).
Rivaroxaban	Patients with stable CVD receiving low-dose rivaroxaban (2.5 mg twice daily) plus aspirin had a 24% relative risk reduction in the composite of cardiovascular death, stroke, or myocardial infarction, compared with aspirin alone.	NA	Adding a second antithrombotic drug (P2Y12 inhibitor or low-dose rivaroxaban) to aspirin for secondary prevention should be considered in patients at high risk of ischemic events and without high bleeding risk (Class IIa).
SGLT2 inhibitors and GLP-1RA	SGLT2 inhibitors and GLP1-RA decrease the incidence of myocardial infarction, stroke, or cardiovascular death by 14% in patients with established ASCVD.^21^^,^^22^	Consider SGLT2 inhibitor or GLP-1RA in patients with type 2 diabetes and established ASCVD.^a^	In persons with type 2 DM and ASCVD, the use of a GLP1-RA or SGLT2 inhibitor is recommended to improve CVD and cardiorenal outcomes (Class I).
Colchicine	23% Relative reduction in ASCVD outcomes with low-dose colchicine in recent acute MI; 28% relative risk reduction in the composite end point of cardiovascular death, MI, or stroke in chronic coronary syndromes.^23^	NA	Low-dose colchicine may be considered in secondary prevention of CVD, particularly if other risk factors are not well controlled or if recurrent events occur under optimal therapy (Class IIb).

ACC/AHA = American College of Cardiology, American Heart Association; ACE = angiotensin-converting enzyme; ARB = angiotensin receptor blocker; ASCVD = atherosclerotic cardiovascular disease; BP = blood pressure; CAD = coronary artery disease; CV = cardiovascular; CVD = cardiovascular disease; ESC = European Society of Cardiology; DM = diabetes mellitus; GLP1-RA = glucagon-like peptide 1 receptor agonist; LDL-C = low-density lipoprotein cholesterol; MACE = major adverse cardiovascular event; MI = myocardial infarction; PCSK9-inhibitor = proprotein convertase subtilisin/kexin type 9 inhibitor; SBP = systolic blood pressure; SGLT2 inhibitor = sodium-glucose cotrasnsporter-2 inhibitor.

a Recommendations from Expert Consensus Decision Pathways, without grade.

In a multicenter registry of patients who underwent coronary artery revascularization outside the context of randomized controlled trials, adherence to GDMT was a more powerful predictor of freedom from major adverse cardiovascular events than whether revascularization was done surgically or percutaneously.^5^ Considering that the ultimate goal of revascularization guidelines is to reduce major adverse cardiovascular events in potential candidates for coronary revascularization, the absence of specific recommendations or therapeutic guidance in the 2021 AHA/ACC/SCAI Coronary Revascularization Guideline on how to optimize preventive pharmacotherapies beyond antiplatelet agents represents an important opportunity for future discussions on the additive value and clinical benefits of secondary prevention when added to revascularization.

## Medical therapy is underused in patients with CAD who undergo revascularization

Despite the well-established beneficial effects of GDMT in patients with known CAD, prescription and adherence to these drugs is well short of ideal.^6^ From the National Health and Nutrition Examination Survey, it is estimated that among the ∼4 million adults diagnosed with angina in the United States, only 67% and 54% take a statin and antiplatelet agent, respectively.^7^ In the COURAGE, BARI-2D (Bypass Angioplasty Revascularization Investigation 2 Diabetes), and FREEDOM (Future Revascularization Evaluation in Patients With Diabetes Mellitus: Optimal Management of Multivessel Disease) trials, only 34% of the enrolled patients achieved a low-density lipoprotein cholesterol (LDL-C) <70 mg/dL at 1 year.^8^ In the recent ISCHEMIA trial, 25% and 50% of patients were not at goal for systolic blood pressure and LDL-C targets at 1 year, respectively.^9^ Of note, the GDMT prescription rate is even lower for patients who undergo coronary artery bypass graft than for those undergo percutaneous coronary intervention, despite the higher overall burden of atherosclerosis in the former.^10^ In this context, ACC/AHA guidelines need to play a more demonstrative role in emphasizing the importance of optimal preventive therapy for all patients with established CAD, whether or not they undergo revascularization.

## GDMT needs to be directed by guidelines

The term GDMT implies that such pharmacologic treatment is clearly outlined in professional society clinical practice guidelines. However, due to the rapidly changing landscape of cardiovascular preventive therapies, guidelines can quickly become outdated in terms of providing actionable evidence-based treatment recommendations. Moreover, the ACC/AHA at present does not have updated guidelines for preventive care across the spectrum of therapeutic targets for patients with established CAD. Instead, there are temporally disparate recommendations pertaining to this population in separate guidelines, such as non-ST elevation acute coronary syndromes (2014), hypertension (2017), and blood cholesterol (2018).

Therefore, there is a compelling need to provide updated, all-encompassing, consensus recommendations on optimal medical therapy that are better integrated across the therapeutic domains of cardiovascular secondary prevention. For this reason, it seems particularly relevant that the recently promulgated ACC/AHA/SCAI revascularization guideline highlights the need for concomitant pharmacologic secondary prevention and lifestyle intervention as the optimal approach to management. There are, of course, important challenges in doing so, specifically the need to balance thoroughness with the length of the document and, on a broader scope, readability. The ACC/AHA has implemented strategies in the guidelines to address this, including a more “user-friendly” format and the use of more summary figures and flow diagrams, with less text.^11^

Finally, clinical practice guidelines should continue ongoing efforts to keep up with the rapidly changing nature of evidence-based practice. To this end, the ACC/AHA has implemented guideline-focused updates aimed at providing more frequent update of key recommendations within guidelines.^11^ This process can be further expedited by updating recommendations as soon as new data are available, a concept which is now being adopted and is termed “living guidelines.” In parallel, there is a need to implement evidence-based strategies directed at increased guideline adoption, such as audit and direct feedback to providers, and educational outreach visits by local opinion leaders to disseminate best evidence practice.^12^^,^^13^

## What constitutes GDMT for patients with known CAD in 2022?

**Figure 1:**
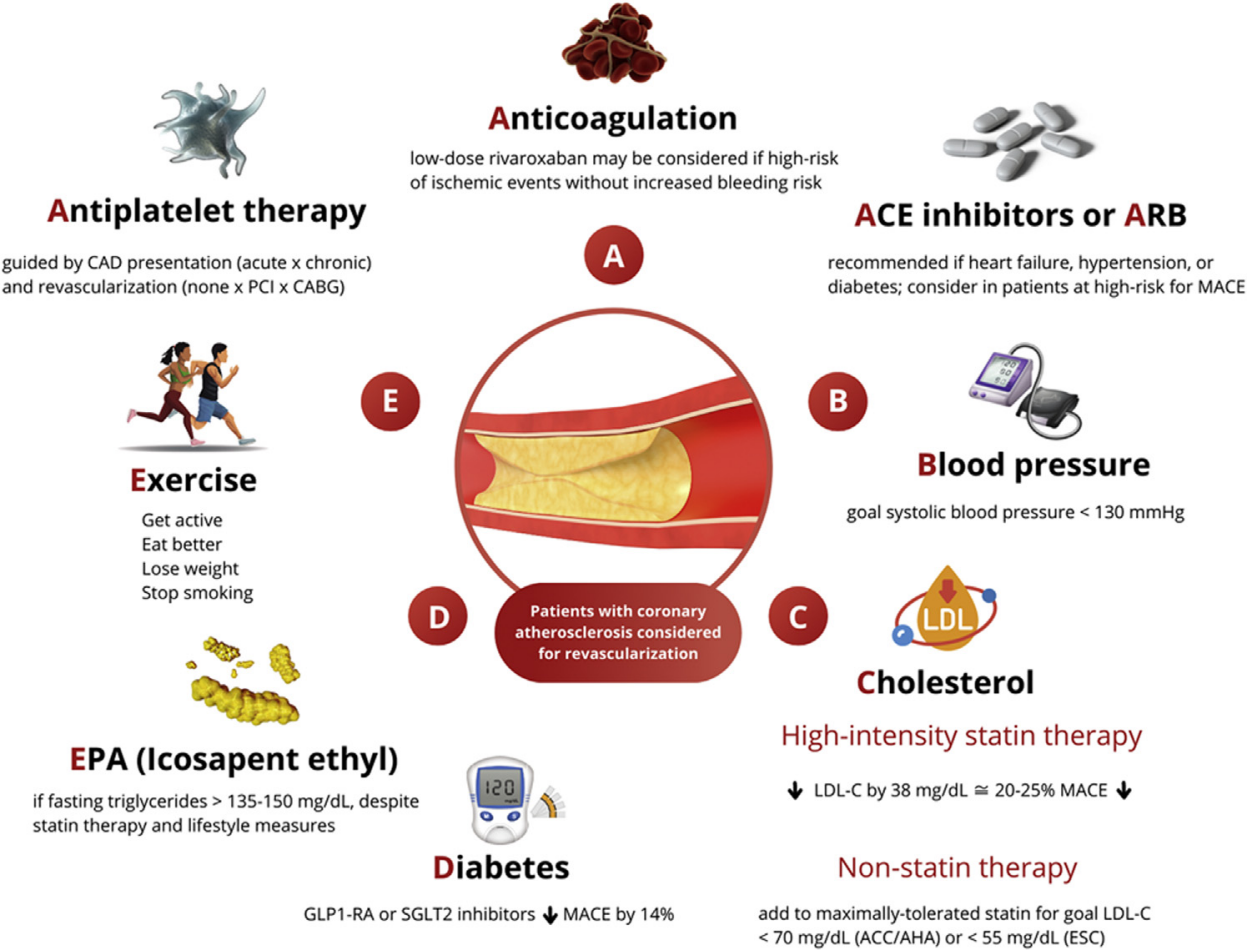
Lifestyle Interventions and Guideline-Directed Medical Therapy for Patients With Established CAD Guideline-directed medical therapy (GDMT) is significantly underutilized in patients who undergo coronary revascularization. Patients with established coronary artery disease (CAD) who are considered for revascularization benefit from implementation of healthy lifestyle changes and GDMT for reduction of atherosclerotic cardiovascular events. Illustration courtesy of Ana Vitória Cordeiro Rocha, Federal University of Goias, Brazil. ACC = American College of Cardiology; ACE = angiotensin-converting enzyme; AHA = American Heart Association; ARB = angiotensin receptor blocker; CABG = coronary artery bypass graft; EPA = ethyl eicosapentaenoic acid; ESC = European Society of Cardiology; GLP1-RA = glucagon-like peptide 1 receptor agonist; LDL-C = low-density lipoprotein cholesterol; MACE = major adverse cardiovascular event; PCI = percutaneous coronary intervention; SGLT2 = sodium-glucose cotrasnsporter-2.

Acknowledging these aforementioned limitations, we propose a model schema that would further advance the 2021 ACC/AHA/SCAI Guideline for Coronary Artery Revascularization by addressing the critical role that multifaceted preventive therapy plays in the overall management of patients being considered for coronary artery revascularization (Figure 1). The rapidly growing arsenal of prevention-related pharmacotherapies now includes antiplatelet therapies, low-dose anticoagulants, statin and nonstatin therapies for lowering LDL-C, icosapent ethyl, and sodium-glucose cotrasnsporter-2 inhibitors and glucagon-like peptide 1 receptor agonists for patients with diabetes or obesity. Table 1 summarizes key evidence about these agents from randomized clinical trials and current guideline-directed recommendations.

In conclusion, optimal medical and lifestyle therapies represent the cornerstone of secondary prevention addressing multiple treatment targets to lower residual cardiovascular risk in patients with stable CAD and in those who recover from an acute atherosclerotic cardiovascular disease event. Despite this compelling therapeutic need, such interventions are significantly underutilized in patients who undergo coronary artery revascularization and for whom such systemic therapy has been already proven convincingly to reduce incident cardiovascular events.

For these reasons, we believe that subsequent modifications and enhancements to these otherwise carefully crafted 2021 ACC/AHA/SCAI Coronary Revascularization Guideline would further underscore the critical importance that concomitant preventive therapies would have in enhancing event-free survival and improving outcomes in patients with known atherosclerotic cardiovascular disease.

## Funding support and author disclosures

Dr Arbab-Zadeh has received grant support from Canon Medical Systems. All other authors have reported that they have no relationships relevant to the contents of this paper to disclose.
